# Supplementary material for: Content-rich biological network constructed by mining PubMed abstracts
Source: BMC Bioinformatics. 2004 Oct 8;5:147. doi: 10.1186/1471-2105-5-147 (PMC528731; doi:10.1186/1471-2105-5-147)
Supplement: Additional File 5 — The original Chilibot query results of the term "long-term potentiation (LTP)" and 22 other terms, limiting the latest references analyzed to the years 1990, 1995, 2000, and 2004. [file 1471-2105-5-147-S5.bz2 › chilibotAdditionalFile5/ltp2004/html/CREB.html]

 


**CREB** (Input: CREB ) 

---


**Google Searches:** Entire Web  | EDU domain only  | PDF files only

.

**External Links:** OMIM |
LocusLink
| Swissprot
| GeneCards

  
**Maps of CREB**

Simple
Complete
graph in
radiant
tree
square
layout.

**New Hypothesis !**

**Synonyms** 

- creb   [PubMed]

**Synopsis**

- **CREB** is a hormonally stimulated transcriptional activator, and CaM kinase may lie on the pathway to its activation.  Annu Rev Biochem, 1992 
  [28]

- These results suggest that several proteins within the C EBP family including ATF4 **CREB** 2 act to constrain long term synaptic changes and memory formation.  Neuron, 2003 
  [23]

- This view is supported by studies on alpha delta **CREB** knockouts, and provides a starting point for studying the role of gene expression in experience dependent cortical plasticity.  Neuroscience, 2002 
  [21]

- These results suggest that NO, cGMP, and PKG cause release of calcium from ryanodine sensitive stores, which in turn causes phosphorylation of **CREB** in parallel with PKA during the induction of L LTP.  J Neurophysiol, 2002 
  [20]

- We show that pharmacological blockade of MAPK ERK phosphorylation, functional inactivation of **CREB** in an inducible transgenic mouse and inactivation of zif268 in a mutant mouse result in a similar deficit in long term recognition memory.  Philos Trans R Soc Lond B Biol Sci, 2003 
  [20]

- In conclusion, the results of mutant mice suggest that the alternation of catecholamine biosynthesis and cAMP signal pathways may play a key role in development of latent learning and morphine dependence, and they furthermore show that the expression of genes mediated by phosphorylated **CREB** may be involved in the development of latent learning and morphine dependence.  Nippon Yakurigaku Zasshi, 2002 
  [20]

- These results demonstrate that Ras transduces signals elicited by a broad range of stimuli to MAP kinase in hippocampal neurons and further suggest that **CREB** phosphorylation depends on multiple pathways.  J Neurosci, 2001 
  [20]

- Nevertheless, they suggest that some experimental forms of plasticity bypass the requirement for **CREB**.  Neuron, 2002 
  [19]

- Furthermore, we found that exercise influenced the activational state of synapsin as well as of **CREB**, by increasing the phosphorylation of these molecules.  Neuroscience, 2004 
  [19]

- A recent study using mice carrying a mutation in a specific tyrosine residue of trkB pinpoints a necessary role for the phospholipase Cgamma pathway in **CREB** activation and LTP maintenance.  Trends Neurosci, 2003 
  [19]

- Pharmacological and two pathway experiments suggest a model in which VP16 **CREB** activates the transcription of CRE driven genes and leads to a cell wide distribution of proteins that prime the synapses for subsequent synapse specific capture of L LTP by a weak stimulus.  Cell, 2002 
  [18]

- The new synapses are enriched in NMDA receptor and it was found that their generation involves activation of NMDA receptors, PKA and **CREB**.  Neurol Neurochir Pol, 2003 
  [18]

- **CREB** mRNA levels increased in proportion to BDNF mRNA levels.  J Neurophysiol, 2002 
  [17]

- This implies either a species specific or regionally restricted role of **CREB** in the brain and or a compensatory upregulation of the cAMP response element modulator CREM and other as yet unidentified transcription factors.  J Neurosci, 2003 
  [16]

- These observations suggest that Alphabeta acts directly on the pathways involved in the formation of late LTP and agents that enhance the cAMP PKA **CREB** signaling pathway have potential for the treatment of AD.  Proc Natl Acad Sci U S A, 2002 
  [16]
